# Stable Au(111) Hexagonal Reconstruction Induced by Perchlorinated Nanographene Molecules

**DOI:** 10.1021/acs.jpcc.4c03812

**Published:** 2024-10-23

**Authors:** Antoine Hinaut, Sebastian Scherb, Xuelin Yao, Zhao Liu, Yiming Song, Lucas Moser, Laurent Marot, Klaus Müllen, Thilo Glatzel, Akimitsu Narita, Ernst Meyer

**Affiliations:** † Department of Physics, University of Basel, Klingelbergstrasse 82, 4056 Basel, Switzerland; ‡ Max Planck Institute for Polymer Research, Ackermannweg 10, 55128 Mainz, Germany

## Abstract

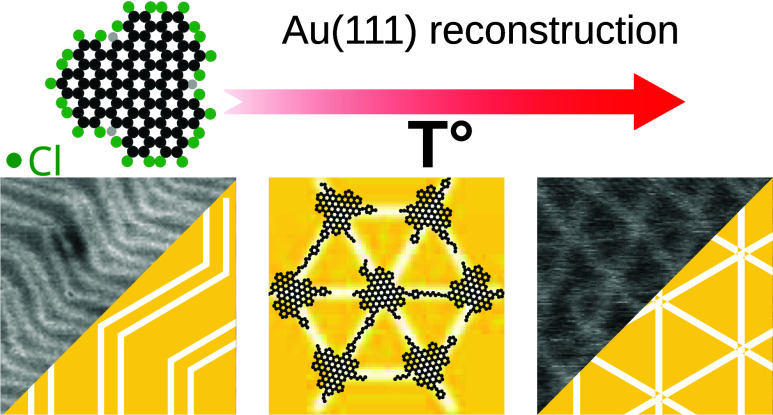

Surface reconstructions play a crucial role in surface science because of their influence on the adsorption and arrangement of molecules or nanoparticles. On the Au(111) surface, the herringbone reconstruction presents favorable anchoring at the elbow sites, where the highest reactivity is found. In this work, we deposited large organic perchlorinated molecules on a Au(111) surface via high-vacuum electrospray deposition. With noncontact atomic force microscopy measurements at room temperature, we studied the molecular structures formed on the surface before and after annealing at different temperatures. We found that a supramolecular layer is formed and that a hexagonal reconstruction of the Au(111) surface is induced. After high-temperature annealing, the molecules are removed, but the hexagonal Au(111) surface reconstruction is preserved. With the hexagonal Au(111) surface reconstruction, a periodic lattice of anchoring sites is formed.

## Introduction

Among existing surface reconstructions, the  herringbone (HB) Au(111) is by far the most well known.^1,2^ The motif already occurs at room temperature and arises when 23 atoms of the surface layer arrange over 22 atoms of the bulk along the [01 1̅] direction in order to minimize surface stress and energy. As a result, a pattern is created on the surface where fcc and hcp domains alternate. Because of the 3-fold symmetry, elbows appear on the surface reconstruction, which are favorable anchoring sites for nanoparticles^3−5^ and molecules.^6−9^

The reactivity of Au(111) can be increased by adjusting the number of available sites on the surface. One possibility is to modify the reconstruction. Atom manipulation and electric field manipulation induced by the tip have shown change of the HB reconstruction in an area up to few hundred nanometers away.^10−12^ The application of external mechanical stress can affect the HB reconstruction to a greater extent.^13^ The deposition of alkali metals can also influence the reconstruction.^14,15^ Also, interfaces formed with adsorbed organic molecules can lead to modifications of the HB reconstruction.^16−21^ In particular, a hexagonal reorganization of the HB reconstruction, the so-called roseta reconstruction,^11,20^ can be found. There, the hcp and fcc domains are still present but are reorganized in a triangular network. Similar reconstructions are predicted for various (111) surfaces as well as for metal layers grown on (111) terminated surfaces.^22^ This hexagonal reconstruction has a higher density of reactive sites, which makes it a good candidate for Au(111) nanostructuring.

A well-known and strong interaction is obtained when a Au surface is exposed to chlorine. Due to the high reactivity, Cl_2_ deposition can lead to a disturbance of the HB reconstruction at low coverage and a complete removal for higher coverages.^23−25^ Chlorine-containing molecules have also shown a high reactivity between Cl and Au atoms, leading to the formation of interesting compounds^26−28^ allowing on-surface molecular templating. In such on-surface processes, Au–Cl bonds are obtained, but no change in the herringbone reconstruction has been reported.

Here, we report the formation of a stable hexagonal reconstruction on the Au(111) surface after the deposition of the molecules functionalized with chlorine and post annealing under ultrahigh-vacuum (UHV) conditions by investigating our samples with atomic force microscopy in the noncontact mode (ncAFM) at room temperature.^29^ Initially, after high-vacuum electrospray deposition^30−33^ (HVESD) of the molecules, densely packed molecular islands are observed. After annealing, the molecules arrange themselves in a hexagonal superlattice. The observed large distance between the molecules indicates the formation of a hexagonal reconstruction at the interface between molecules and the Au(111) surface. The removal of the molecular layer by annealing at a higher temperature and then by classical cycles of sputtering and annealing leads to the formation of a clean and stable hexagonal reconstruction of Au(111). We propose that the molecule composition with 27 chlorine atoms and the observed supramolecular network may lead to the formation of a hexagonal reconstruction of Au(111).

## Method

### Sample Preparation

We used 3 different Au(111) single crystals (Mateck GmbH) prepared in UHV conditions with several cycles of Ar^+^ sputtering and annealing at 750 K. Surfaces are imaged after clean preparation, and herringbone reconstruction was observed for all surfaces in different areas.

### Molecule Synthesis

C_96_H_3_Cl_27_ was prepared following reported procedures.^34^

### Room-Temperature AFM

Room-temperature ncAFM measurements were performed with a home-built noncontact atomic force microscope operated with a Nanonis electronic RC5. PPP-NCL cantilevers (Nanosensor) were used as sensors (typical resonance frequencies of *f*_1_ = 150 kHz, *f*_2_ = 1 MHz, *f*_*t*_ = 1.6 MHz, and oscillation amplitudes *A*_1_ = 2–5 nm, *A*_2_ = 400–800 pm, and *A*_t_ = 40–80 nm for first and second torsional eigenmodes, respectively). Cantilever preparation consisted of annealing for 1 h at 400 K followed by Ar^+^ sputtering for 90 s at 700 eV. During experiments, the base pressure of the UHV system was 2.0 × 10^–11^ mbar. Multipass imaging was acquired following the procedure explained in refs (35,36)(35,36).

### High-Vacuum Electrospray Deposition

The electrospray deposition was performed on samples kept at room temperature using a modified commercial system from Molecularspray, as already explained in refs (37−40). The C_96_Cl_27_H_3_ molecules were dissolved in a toluene:methanol mixture (ratio 5:1). During the spray deposition, the pressure increased to 5.0 × 10^–8^ mbar. The typical applied voltage was 1.2 kV for 10 min.

## Results and Discussion

### Island Formation after Deposition

**Figure 1:**
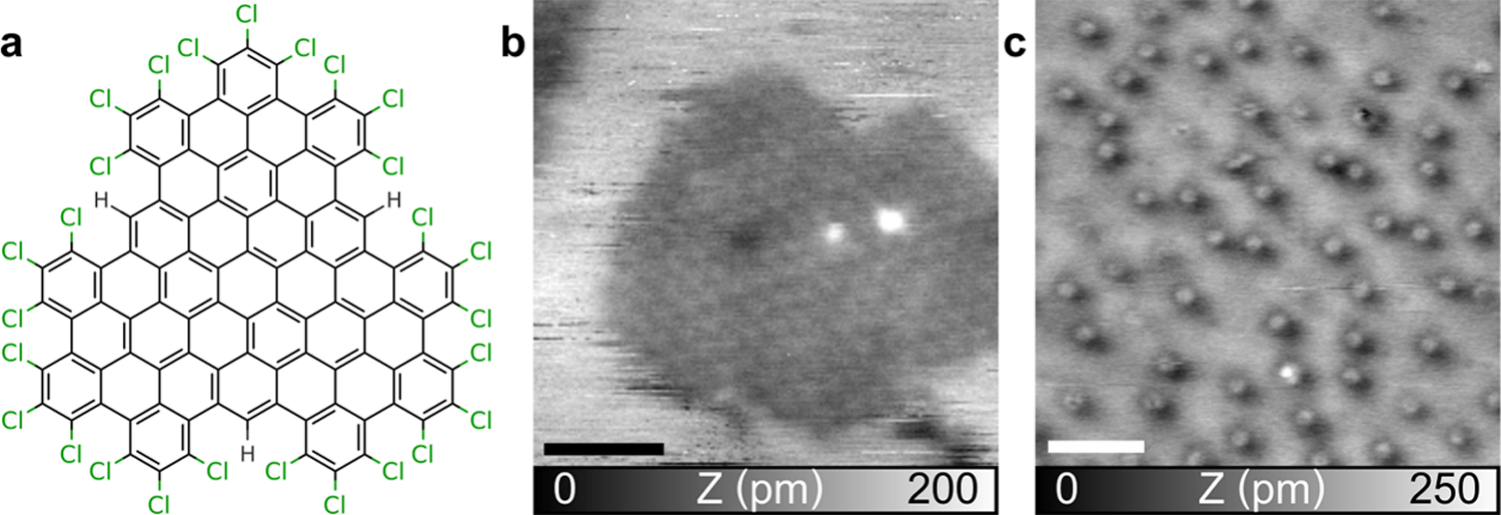
C_96_Cl_27_H_3_ molecules on Au(111). (a) Structure of the C_96_Cl_27_H_3_ molecule. (b) Topography ncAFM image of the C_96_Cl_27_H_3_ island on the Au(111) surface after HVESD. Parameters: *f*_2_ = 1.088 MHz, Δ*f*_2_ = −30 Hz, and *A*_2_ = 800 pm. Scale bar: 5 nm. (c) NcAFM topography on C_96_Cl_27_H_3_ after 350 K annealing. Parameters: *f*_1_ = 167 kHz, Δ*f*_1_ = −40 Hz, and *A*_2_ = 2 nm. Scale bar: 10 nm.

The nanographene molecule,^34^ C_96_Cl_27_H_3_, is composed of 96 sp^2^-hybridized carbon atoms arranged in a triangular shape, with the structure shown in Figure 1a. At the periphery, 27 Cl and 3 H atoms are disposed, resulting in a molecular diameter of about 2 nm. The molecule was already used as a graphene quantum dot in a light experiment study.^41^

The molecules are deposited on the clean Au(111) surface at room temperature via HVESD.^37−40,42−44^ After HVESD, C_96_Cl_27_H_3_ forms regular islands on the Au(111) surface, as visible in the noncontact atomic force microscopy (ncAFM) topography image shown in Figure 1b. The islands have a size up to 50 nm and are displayed with a darker contrast compared to the surface. Individual molecules are distinguished on the island as slightly brighter spots. They form a hexagonal network with a lattice parameter of 2.2 nm, in good agreement with the size of a single molecule. This suggests a dense packing of the molecules on the island. Around the islands, visible with the bright contrast, is the Au surface area, probably covered with some residual mobile molecules at room temperature, e.g., the solvent from the HVESD deposition, as indicated by the horizontal lines with spikes visible in Figure 1b. Such a contrast inversion was already reported for ncAFM imaging^45^ and thus explained by a crossing point in the corresponding Δ*f*(*z*) curves because of chemically different sample sites.

A dispersed phase with well-separated molecules is obtained after an annealing of the surface at 350 K, as visible in the ncAFM topography shown in Figure 1c. There, individual molecules are observed as dots with a diameter of 2.2 nm. The densely packed hexagonal islands and residual mobile species around them are no longer observed. An average distance to the closest neighbor of 8.1 nm is determined between molecules using a 2D autocorrelation function (2D-ACF). A larger-scale image and further details are displayed in Figure S1. Such a long-range separation between molecules can be obtained in cases where interface dipoles are formed upon molecular adsorption^46,47^ inducing repulsion of the molecules.

It is already reported that the C–Cl bond can easily undergo cleavage on surfaces like Au(111) and Ag(111).^26−28^ Thus, the formation of a long-range molecular order could be induced by the presence of the Cl atoms dissociating from the molecules during the annealing process. Their presence on the surface, after cleavage from the molecules, already for the lowest-temperature annealing, could modify the interface dipole between molecules and the surface and induce the largely separated molecular structures. Additionally, Cl is well known to be highly reactive with gold surfaces. Partial to full lifting of the HB reconstruction as well as a Au–Cl surface layer can be obtained after exposure of the sample to Cl.^23−25,48^ Such a surface modification facilitates the formation of a sparse disordered molecular layer.

### Molecular Network and Surface Reconstruction at 450 K

**Figure 2:**
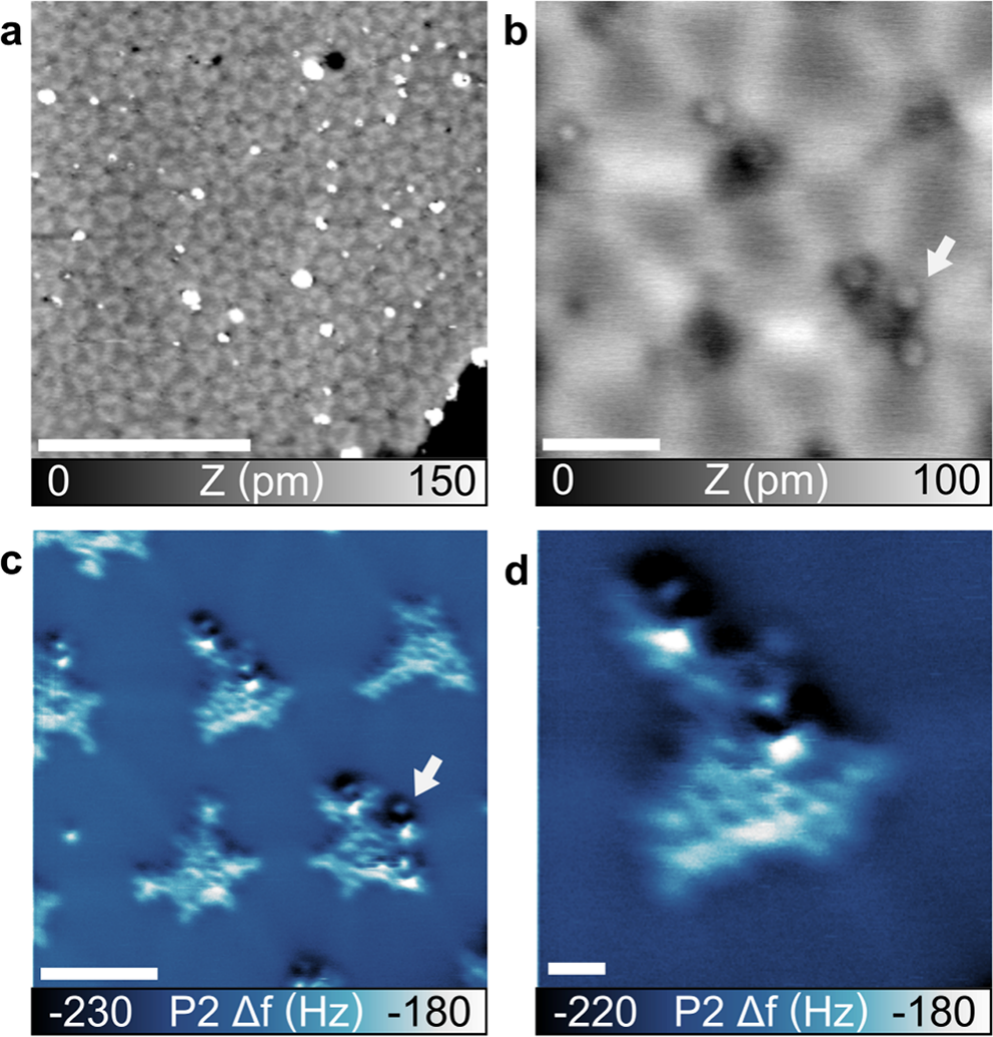
C_96_Cl_27_H_3_ structure after annealing at 450 K. (a) Large-scale ncAFM topography image. Parameters: *f*_1_ = 167 kHz, Δ*f*_1_ = −100 Hz, and *A*_1_ = 2 nm. Scale bar: 50 nm. (b) Zoomed ncAFM topography image and (c) simultaneous second pass frequency shift image. The arrow indicates possible Cl atoms. (d) Second pass frequency shift image on a single molecule; see the SI, Figure 2 for the topography and dissipation. Parameters: *f*_1_ = 167 kHz, Δ*f*_1_ = −200 Hz, and *A*_1_ = 2 nm; second pass P2 Δ*Z* = −150 pm. Scale bar for panels (b, c): 5 nm and for panel (d): 1 nm.

A hexagonal pattern is observed on the Au(111) surface after annealing at 450 K, as visible in the topography image shown in Figure 2a. The structure is extended over hundreds of nanometers covering all of the terraces; see Figure S2 for a larger overview of the area. The zoomed-in topography image reveals a better view of the structure, as seen in Figure 2b. The pattern is composed of dark areas, surrounded by six extended protrusions, forming a hexagonal structure. Triangular areas are formed by three bright lines and a distance of 7.5 nm is measured between dark spots.

The corresponding second pass frequency shift image^35,36^ allows identifying single molecules in the network, as visible in Figure 2c. The molecules are positioned on the areas corresponding to the dark spot of the topography image; also see Figure S3 for additional signals helping in molecule identification. Although the triangular shape is partially preserved for the molecules, they all present different randomly extended structures with sizes up to 4 nm, i.e., larger than the molecular dimensions. The molecule extensions are aligned along the bright protrusion of the network, as seen in the topography images of Figure 2b and SIFigure 2a, suggesting a growth orientation driven by the hexagonal structure of the surface. Most probably, such elongated structures are carbon-based chains and fragments that derive from the C_96_Cl_27_H_3_ molecules and the eventual remaining solvent. A single molecule is visible in the second pass frequency shift image shown in Figure 2d with different extension structures (also see Figure S4 for other signals). Distorted rings are also visible in the center of the molecule, possibly a sign of the nanographene-like structure.

As visible in the second pass frequency shift image shown in Figure 2c, an empty space of around 2–3 nm is measured in between the molecules. Such a lattice dimension of 7.5 nm is much larger than the lattice measured for the densely packed island before annealing, as shown in Figure 1b, and much larger than typical molecular interactions.^47^ The network is also smaller than the average nearest neighbor distance measured after annealing at 350 K.

The observed hexagonal pattern of the Au(111) surface, as observed in the topography images of Figure 2a,b (and also in the magnified contrast image of Figure S3d), is due to a hexagonal reconstruction on the Au(111) surface. Such a reconstruction, also called roseta, was already observed.^11,20,49^ The lifting of the herringbone reconstruction and its replacement by the roseta induce the positioning of the molecules and the formation of the superlattice. There, the intersection of the hills in the roseta reconstruction acts as favorable anchoring sites for the molecules.^9^

The variation in the extension of the extended structures can be explained via the coupling reaction of the molecules. Although the carbon–halogen bond cleavage can explain the sparse network observed for low-temperature annealing, here the higher temperature induces polymerization. Additional protrusions (see arrows) are observed in the topography and second pass frequency shift images shown in Figure 2b,c (and in SI Figures 3 and 4) and could possibly be attributed to some Cl atoms.

### Molecular Network Structure Evolution at 550 K

**Figure 3:**
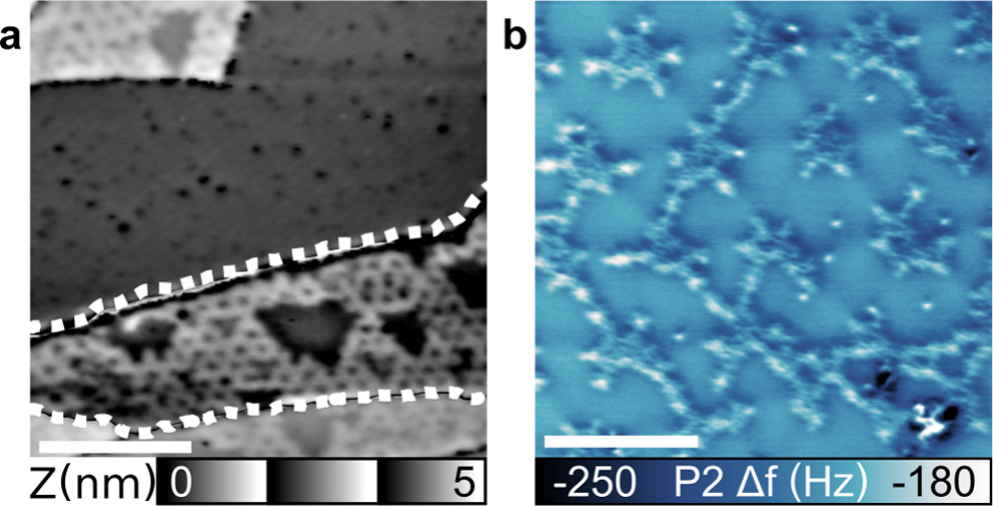
C_96_Cl_27_H_3_ structure after 550 K annealing. (a) ncAFM topography of the hexagonal structure. A specific gray color scale is used. (b) Second pass frequency shift zoomed image on a structure obtained after 550 K annealing. Corresponding topography and dissipation images are displayed in the SI. Parameters: *f*_1_ = 167 kHz and *A*_1_ = 2 nm, for panel (a): Δ*f*_1_ = −25 Hz, and for panel (b): second pass Δ*Z* = −200 pm. Scale bar for panel (a): 50 nm and for panel (b): 10 nm.

The superlattice is partially preserved on Au(111) after annealing at 550 K, as visible in the topography image of Figure 3a. There, large areas of the surface exhibit the previously observed hexagonal structure with embedded triangular-shaped holes, not observed before. Similar to Figure 1, because of the inverted contrast during ncAFM measurement, the location of the molecules corresponds to the dark holes. Large domains of the surface are not covered with the superlattice, as visible in the upper part of the Figure 3a image. There, no HB reconstruction is imaged, and some protrusions and extended lines are observed (see the SI Figure 5).

**Figure 4:**
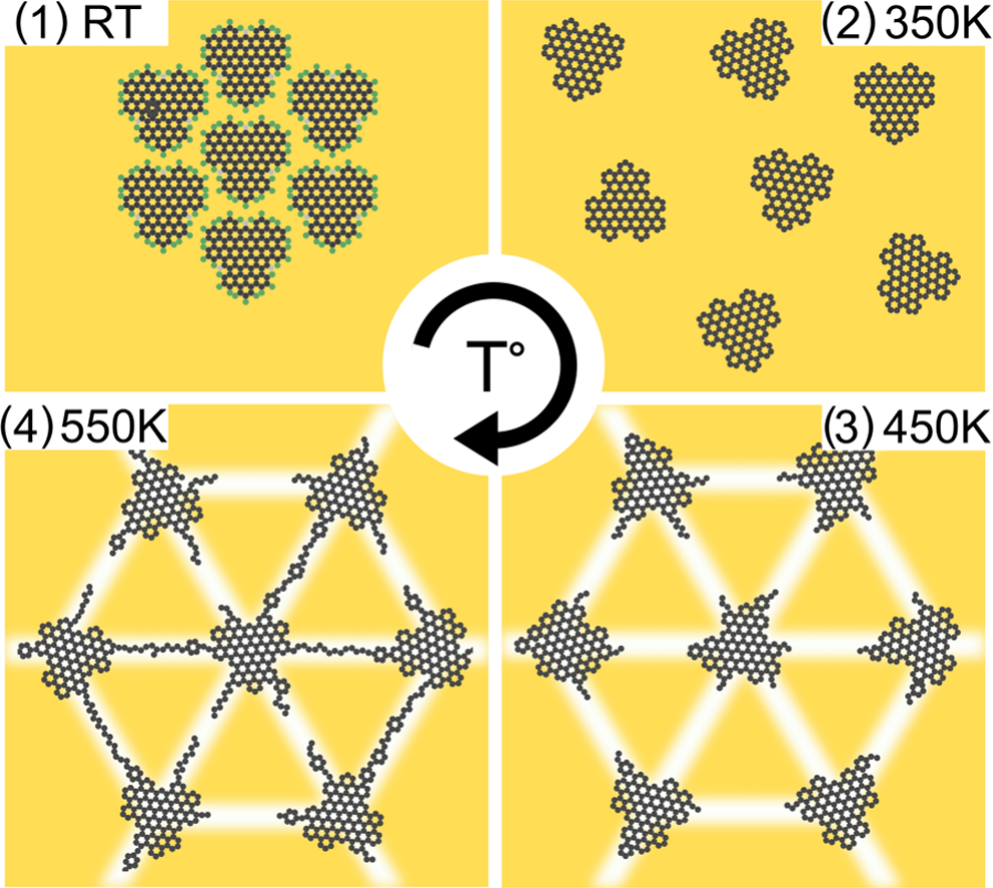
Scheme of the molecular network after successive annealing steps: (1) before and after (2) 350, (3) 450, and (4) 550 K annealing.

While the superlattice dimensions are preserved for the 550 K annealing, the second pass frequency shift image of Figure 3b reveals the formation of carbon-based chains interconnecting the molecules. Especially, molecules are still present at their specific hexagonal sites (also see the SI, Figure 4) and start to be interconnected with chains formed along the bright protrusion pathway of the surface (similar to the ones shown in Figure 2a). Upon increasing the polymerization, some of the molecules are used as the carbon source. Therefore, molecules can locally be removed to builtd up molecular chains, leading to the formation of triangular holes and the appearance of a superlattice.

### Discussion on the Evolution with Temperature

The situation before and after the different annealing steps is schematically represented in Figure 4. After the HVESD deposition, the molecules form densely packed islands, obtained at room temperature (RT). The first step of annealing at 350 K induces a large separation distance between molecules, without a highly ordered network. Interestingly, already with this gentle annealing, the herringbone reconstruction of the Au(111) surface is not observed anymore. After the second-step annealing, at 450 K, a roseta reconstruction of the Au(111) surface is observed, promoting the formation of a long-range hexagonal molecular superlattice structure. Additionally, the molecules present different extended shapes aligned along the hexagonal reconstruction. The superlattice presents dimensions of 7.5 nm, higher than the molecular network before annealing but smaller than the average distance to the first neighbor measured in the sparse network for the first annealing step. Finally, at 550 K annealing, the chain structures interconnecting the molecules are formed aligned along the hexagonal reconstruction of the surface, forming an interconnected carbon-based molecular network.

### Clean Au(111) and Hexagonal Reconstruction

**Figure 5:**
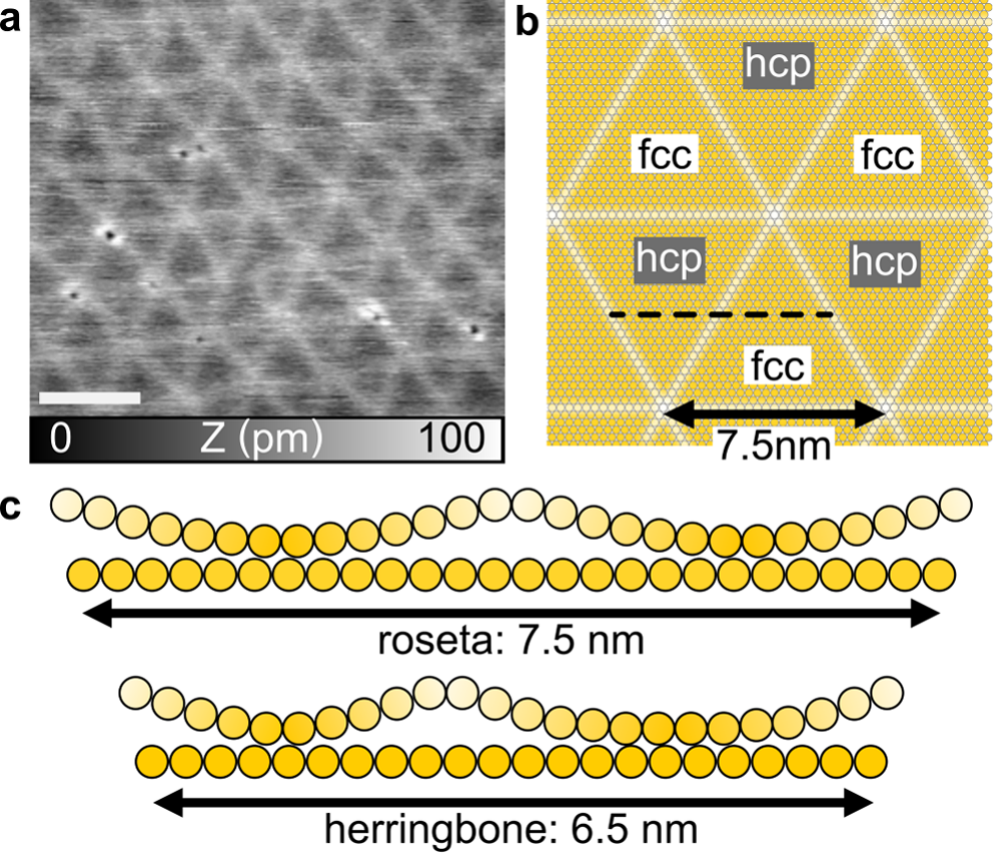
Hexagonal reconstruction on the bare Au(111) surface. (a) ncAFM topography image of the Au(111) hexagonal reconstruction. (b) Top view of the proposed hexagonal reconstruction. (c) Comparative side view of the hexagonal and herringbone reconstruction. Parameters: *f*_1_ = 166 kHz, Δ*f*_1_ = −80 Hz, and *A*_1_ = 2n m. Scale bar: 10 nm.

A highly nanostructured Au(111) surface is obtained by removing the supramolecular structure by 800 K annealing, as visible in Figure 5a. A closer look at the structure shows that the intersection between the extended protrusions is no longer dark (see Figure 5b), which indicates that the molecules are completely removed. We also performed X-ray photoelectron spectroscopy (XPS) measurement on the sample to confirm the removal of the Cl species.^23^ As visible in Figure S7, no trace of Cl is observed. This absence is intriguing since even without molecules and Cl, the hexagonal reconstruction is observed; see Figure S8 for the large-scale area of the surface. Performing further cleaning cycles of Ar^+^ sputtering and annealing on the sample did not fully remove the hexagonal reconstruction. Using XPS, we could not evidence any influence of bulk contamination of our sample, which could promote the reconstruction via their diffusion to the surface during annealing (see Figure S9).

The distance of 7.5 nm measured between intersections, equal to the separation distance of C_96_Cl_27_H_3_ on the Au(111) surface, is reported and shown in Figure 2c,d. Such a distance suggests a Au(111) 27 × 27 hexagonal reconstruction, larger than the HB reconstruction, as seen with the model shown in Figure 5b,c. The motif would be composed of fcc and hcp domains of the same size and organized in hexagonal lattices. On the sample, the intersection between domains appears to be reactive sites, as they often are contaminated (see white arrows in Figure 5a). Such a hexagonal reconstruction could even increase the density of reactive sites compared to the HB reconstruction.

## Conclusions

We formed a stable hexagonal surface reconstruction on Au(111) by the deposition of a perchlorinated nanographene molecule and various annealing steps. At first, dense islands with molecules are formed on the Au(111) surface. A first annealing step promotes the halogen–carbon bond cleavage and leads to the formation of a sparse disorganized molecular layer on Au(111). The second annealing step promotes the creation of a hexagonal surface reconstruction of Au(111) together with the creation of a molecular network showing a superlattice aligned to the hexagonal Au(111) reconstruction. With further annealing, the molecules become interconnected with carbon chains grown along the hexagonal surface reconstruction. Higher-temperature annealing of the surface leads to the removal of the molecules. The Au(111) hexagonal reconstruction with a periodicity of 7.5 nm is still observed, indicating a very stable state. Such reconstructed surfaces present a high density of reactive sites directly on the metal surface and could therefore be used for nanotemplating molecules or nanoparticles with potential applications in catalysis.

## Supporting Information Available

Part 1: Characterization (ncAFM) of the molecular network after 350 K annealing (Figure S1).
Part 2: Characterization (ncAFM) of the molecular network after 450 K annealing (Figures S2, S3, and S4).
Part 3: Characterization (ncAFM) of the surface after 550 K annealing (Figures S4 and S6).
Part 4: Characterization (XPS and ncAFM) of the surface after the removal of the molecules (Figures S7, S8, and S9).
