# Supplementary material for: Stable Au(111) Hexagonal Reconstruction Induced by Perchlorinated Nanographene Molecules
Source: J Phys Chem C Nanomater Interfaces. 2024 Oct 23;128(44):18894–900. doi: 10.1021/acs.jpcc.4c03812 (PMC11552072; doi:10.1021/acs.jpcc.4c03812)
Supplement: Supplementary file 1 — jp4c03812_si_001.pdf [file jp4c03812_si_001.pdf]

Supplementary Information: Stable Au(111) Hexagonal Reconstruction Induced by Perchlorinated Nanographene Molecules

Antoine Hinaut,† Sebastian Scherb,† Xuelin Yao,‡ Zhao Liu,† Yiming Song,† Lucas Moser,† Laurent Marot,† Klaus Müllen,‡ Thilo Glatzel,† Akimitsu Narita,‡ Ernst Meyer*†*

†Department of Physics, University of Basel, Klingelbergstrasse 82, 4056 Basel, Switzerland
‡Max Planck Institute for Polymer Research, Mainz, Germany

E-mail: antoine.hinaut@unibas.ch; ernst.meyer@unibas.ch

Part 1: Sparse molecular network on Au(111) after 350 K annealing.

Profiles on individuals molecules shows a ~2,2nm diameter (SI Figure 1ab).
A different contrast ncAFM topography is visible on the SI Figure 1c. Using 2D ACF (SI Figure 1de), an average distance of 8,1 nm is measured between molecules.

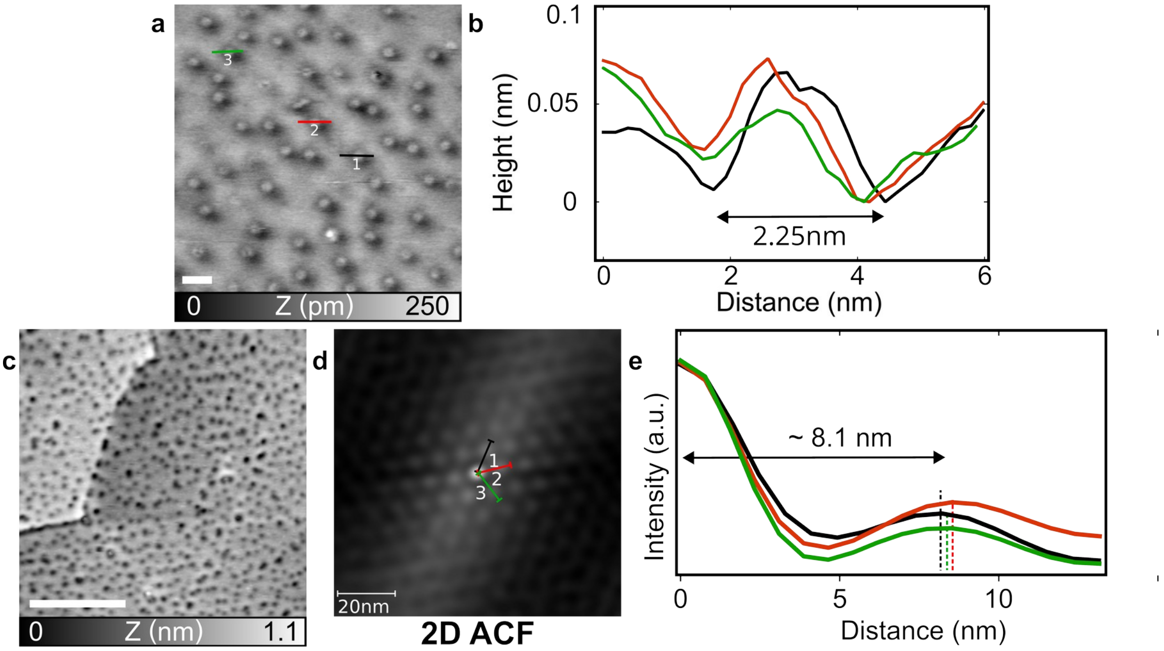

Figure S1: Network after 350K annealing. a) ncAFM topography image. b) Profiles over individual molecules. c) Larger scale ncAFM topography with a different contrast on the same area. d) corresponding 2D autocorrelation function. e) Profiles from d). Parameters: f_1=167kHz, A_1=2nm. a) Δf_1=-40Hz, c) Δf_1=-100Hz. Scale bar: a) 5nm. c) 50nm.

Part 2: Organized Molecular network on Au(111) after 450 K annealing.

The ncAFM topography image in SI Figure 2 shows the extension of the molecular network over distances > 100nm.
Contrast is modified to reveal structure on the different step edges.

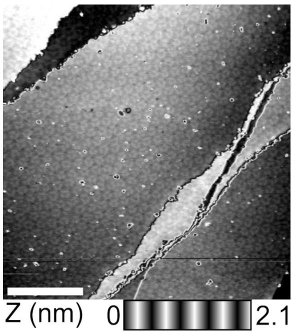

Figure S2: ncAFM topography. Parameters: f_1=167kHz, Δf_1=-100Hz, A_1=2nm. Scale bar: 50nm.

Superposing the molecular contour from the frequency shift second pass image on the topography help to reveal the alignment of the molecules towards the Au(111) surface reconstruction (SI Figure 3a).
Using simultaneously acquired dissipation (SI Figure 23) and second pass torsional imaging (SI Figure 3c) confirm the positioning of the molecules.
The surface reconstruction is also visible in the second pass frequency shift image using a modified contrast (SI Figure 3d).

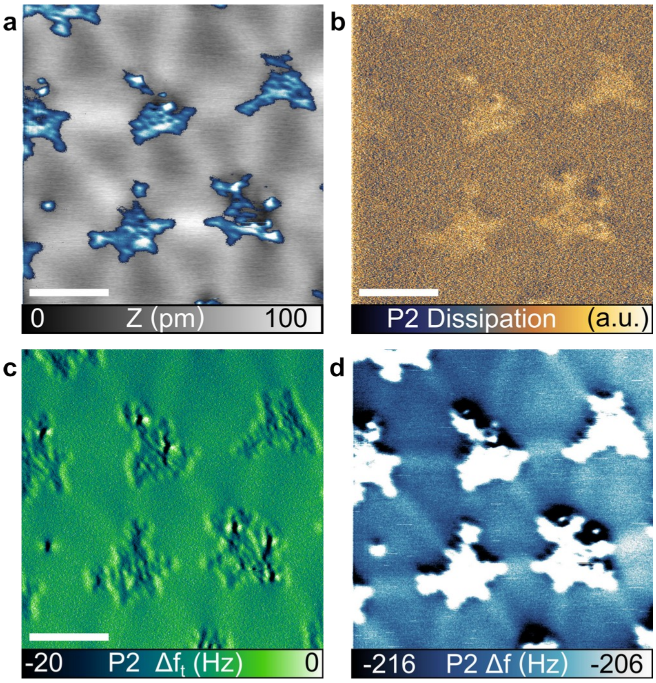

Figure S3: ncAFM of the molecular network after 350 K annealing. a) Superposition of the molecules in frequency shift second pass from Figure 2c on the topography image, Figure 2b . Corresponding b) dissipation and c) torsional frequency shift of the second pass scan. d)

magnified contrast of second pass frequency shift image. Parameters: f_1=167kHz, Δf_1=-200Hz, A_1=2nm, f_t=1.51MHz, A_t=80pm, Second pass P2-ΔZ= -150pm. Scale bar: 5nm.

The dark contrast area of the topography image (SI Figure 4a) correspond to the position of the single molecules from Figure 2d. The single molecule is well observed in the second pass dissipation (SI Figure 4b) and torsionnal frequency shift (SI Figure 4c) images.
The round shape like protrusion (also in Figure 2bc) are also visible around this single molecule as indicated by the arrows on every image.

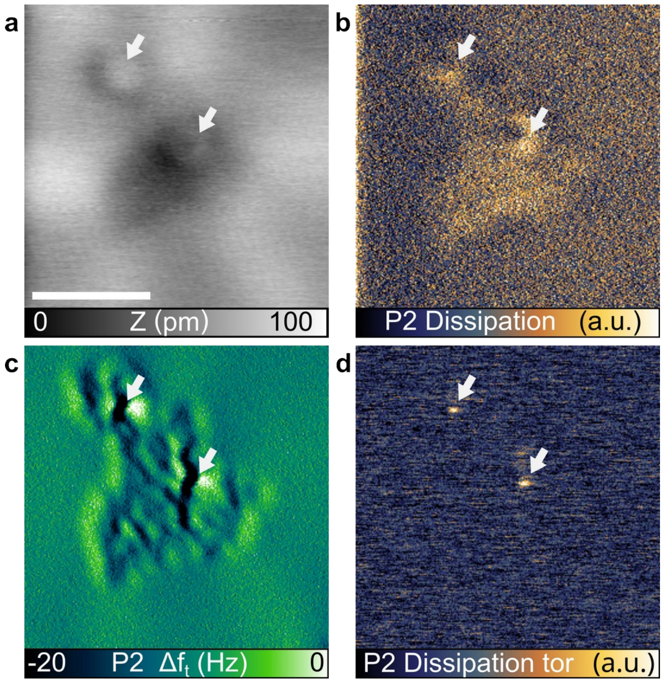

Figure S4: ncAFM on a single molecule after 350 K annealing. a) Topography. Second pass b) dissipation, c) torsional frequency shift and d) torsional dissipation. Parameters: f_1=167kHz, Δf_1=-200Hz, A_1=2nm, f_t=1.51MHz, A_t=80pm, Second pass P2-ΔZ= -150pm. Scale bar: 3nm.

Part 3: After 550 K annealing.

Au(111) areas without the hexagonal reconstruction present a lot of defects and extended rows structures as visible in the topography (SI Figure 5 a) and corresponding profile (SI Figure 5 B). The rows separation is 5 nm and can not be attributed to herringbone reconstruction, as visible in the profile.

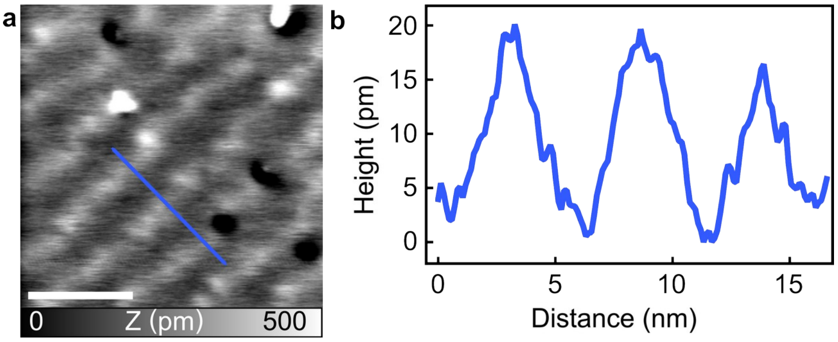

Figure S5: Au(111) unreconstructed area. ncAFM topography and corresponding Excitation images. Parameters: f_1 = 167kHz, Δf_1 = -200Hz, A_1 = 2nm.

First pass topography ncAFM and dissipation images corresponding to the second pass frequency shift of figure 3 b. Similar to the 450 K annealing, the molecular superlattice is only visible as darker areas in the topography image. But molecules present a contrast in the dissipation image compared to the Au(111) surface.

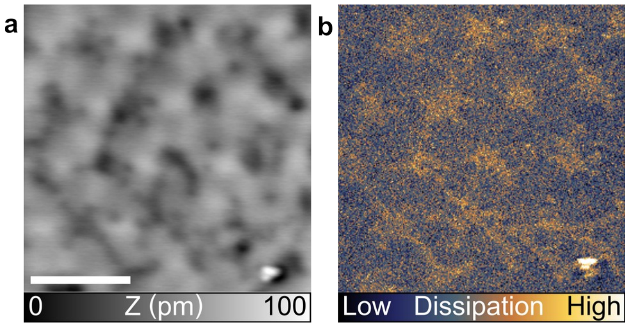

Figure S6: ncAFM topography and corresponding Excitation images. Parameters: f_1 = 167kHz, Δf_1 = -200Hz, A_1 = 2nm.

Part 4: Roseta reconstructed Au(111) sample.

To confirm the absence of remaining chlorine on the Au surface, we performed XPS measurements. SI Figure 7 presents the core level XPS spectra of the samples. The survey measurement (SI Figure 7a) using a pass energy of 50 eV. A zoom of the region 80-240 eV is presented in SI Figure 7a). Au4f and C1s core level spectra are presented in SI Figure 7b,c. Cl2p region was measured with a pass energy of 29 eV and using 60 scans (SI Figure 7d). No chlorine was revealed.

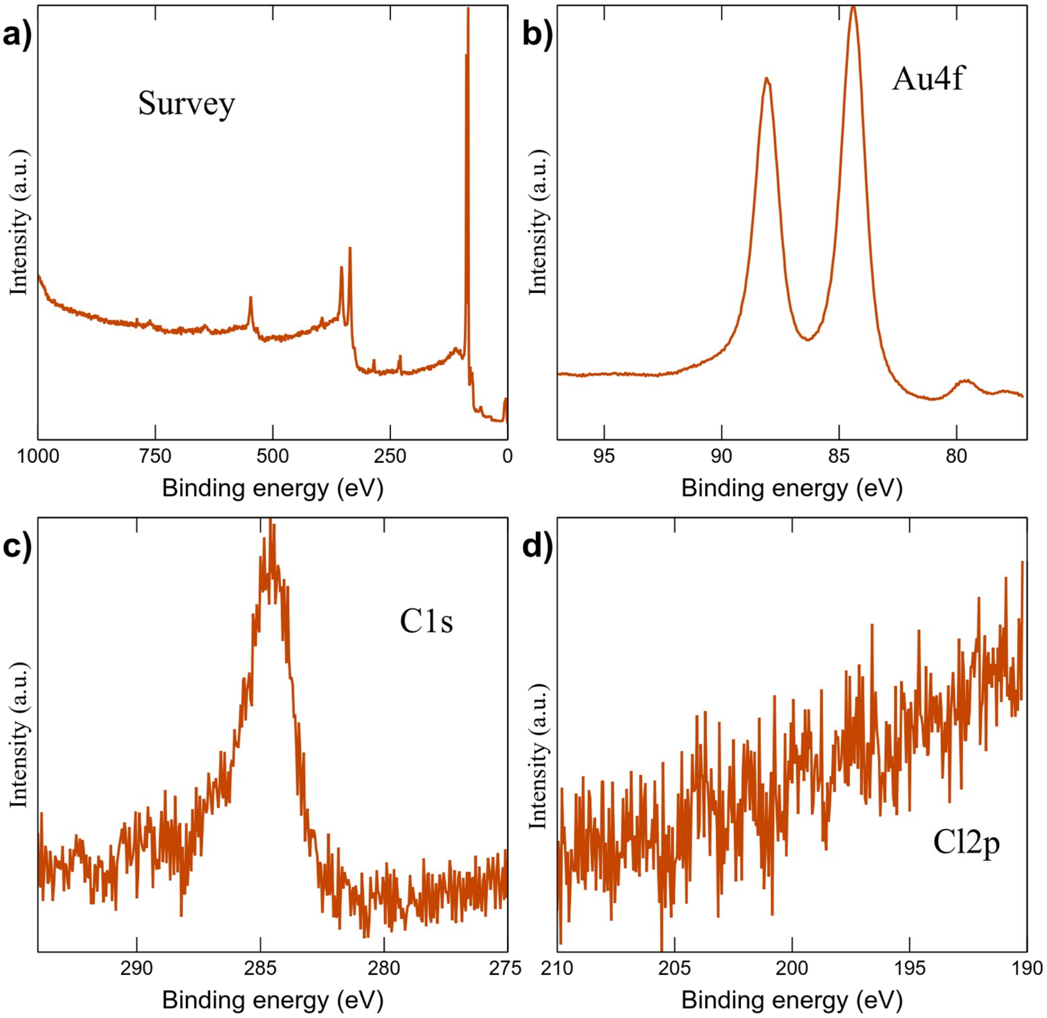

Figure S7: XPS measurement on the Au(111) clean sample with roseta reconstruction. a) Survey, b) Au4f, c) C1s and d) Cl2p.

X-ray photoelectron spectroscopy (XPS) was carried out using electron spectrometer equipped with a hemispherical analyzer (Leybold EA10/100 MCD) and a non-monochromatized magnesium Kα X-ray source (hν = 1253.6 eV). The binding energy scale was calibrated using the $Au4f_{7/2}$ line of a cleaned Au sample at 84.0 eV. The acquisition mode was set to constant analyzer energy with 29 eV pass energy (0.05 eV step size) and normal electron escape angle. The typical resolution is 0.8 eV.

After several cycle of Ar sputtering and cleanning, the roseta reconstruction is still observed on the Au(111) surface. As visible in the SI Figure 8a, large domain, covering half of the

surface are observed presenting the hexagonal structure. The corresponding dissipation image helps to discriminate the areas.
The hexagonal area have different appearance with respect to the Au(111) surface a vsisble with contrast change in the image, indicated with dotted lines.

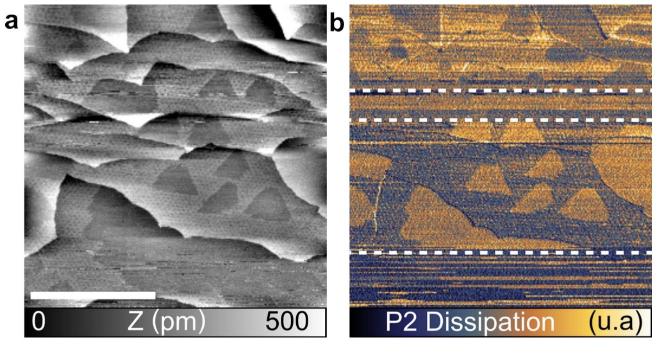

Figure S8: ncAFM topography a) and corresponding Excitation b) images. Parameters: f_1 = 164kHz, Δf_1 = -23Hz, A_1 = 5nm.

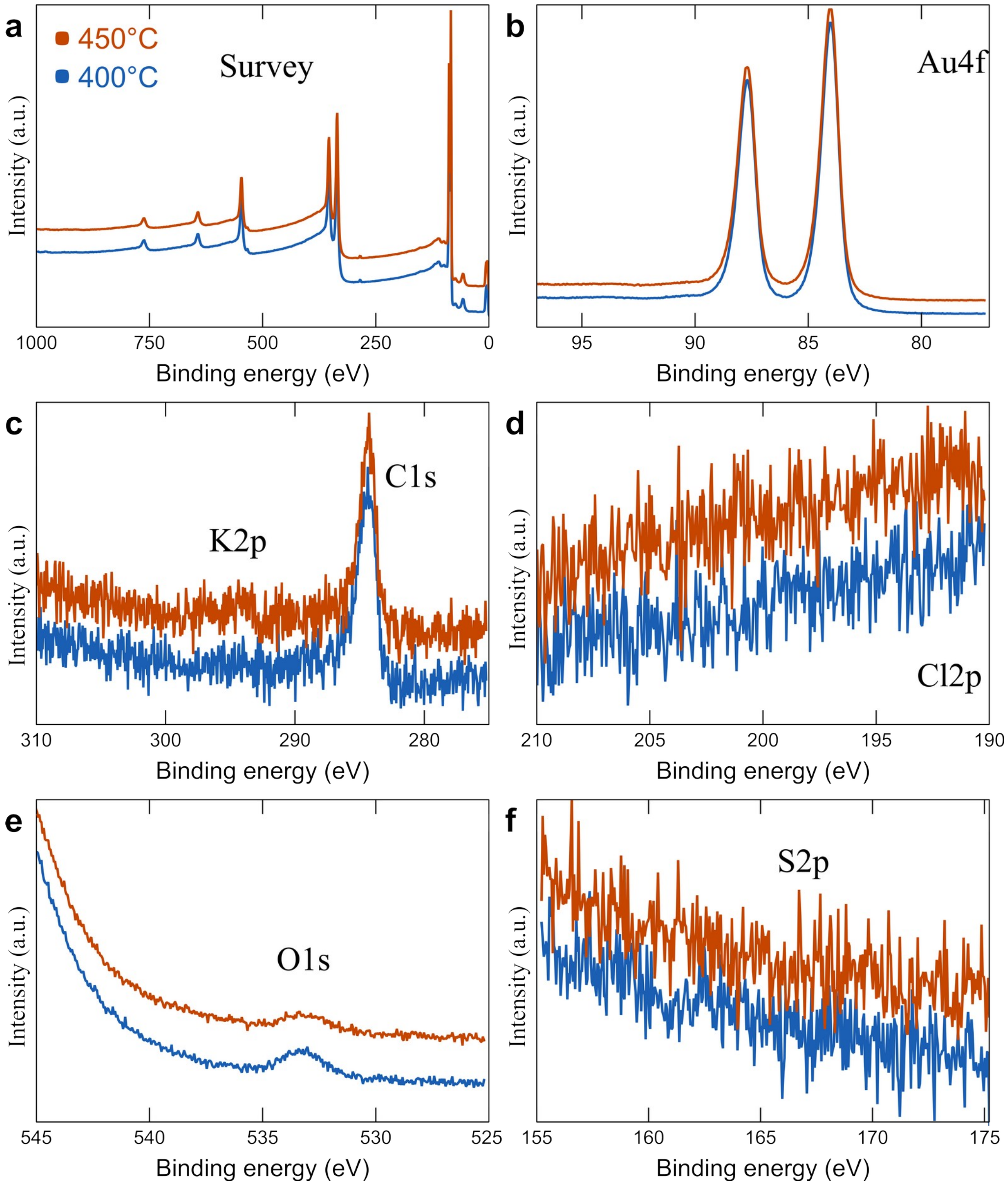

Figure S9: XPS measurement on the Au(111) after annealing at 400°C (blue) and 450°C (red). a) Survey, b) Au4f, c) C1s and K2p, d) Cl2p, e) O1s and f) S2p.

References :

[1] David Nečas, Petr Klapetek, Gwyddion: an open-source software for SPM data analysis, *Cent. Eur. J. Phys.* **10**(1) (2012) 181-188

[2] Rahe, P.; Bechstein, R.; Schütte, J.; Ostendorf, F.; Kühnle, A. Repulsive Interaction and Contrast Inversion in Noncontact Atomic Force Microscopy Imaging of Adsorbates. Physical Review B 2008, 77, 195410.
